# Metabotropic Glutamate Receptor-1 as a Novel Target for the Antiangiogenic Treatment of Breast Cancer

**DOI:** 10.1371/journal.pone.0088830

**Published:** 2014-03-14

**Authors:** Cecilia L. Speyer, Ali H. Hachem, Ali A. Assi, Jennifer S. Johnson, John A. DeVries, David H. Gorski

**Affiliations:** 1 Tumor Microenvironment Program, Barbara Ann Karmanos Cancer Institute, Wayne State University School of Medicine, Detroit, Michigan, United States of America; 2 Molecular Therapeutics Program, Barbara Ann Karmanos Cancer Institute, Wayne State University School of Medicine, Detroit, Michigan, United States of America; 3 Department of Surgery, Wayne State University School of Medicine, Detroit, Michigan, United States of America; 4 University of Michigan, Dearborn, Michigan, United States of America; 5 Van Andel Research Institute, Grand Rapids, Michigan, United States of America; Florida International University, United States of America

## Abstract

Metabotropic glutamate receptors (mGluRs) are normally expressed in the central nervous system, where they mediate neuronal excitability and neurotransmitter release. Certain cancers, including melanoma and gliomas, express various mGluR subtypes that have been implicated as playing a role in disease progression. Recently, we detected metabotropic glutamate receptor-1 (gene: *GRM1*; protein: mGluR1) in breast cancer and found that it plays a role in the regulation of cell proliferation and tumor growth. In addition to cancer cells, brain endothelial cells express mGluR1. In light of these studies, and because angiogenesis is both a prognostic indicator in cancer correlating with a poorer prognosis and a potential therapeutic target, we explored a potential role for mGluR1 in mediating endothelial cell (EC) proliferation and tumor-induced angiogenesis. *GRM1* and mGluR1 were detected in various types of human ECs and, using mGluR1-specific inhibitors or shRNA silencing, we demonstrated that EC growth and Matrigel tube formation are dependent on mGluR1 signaling. In addition, loss of mGluR1 activity leads to reduced angiogenesis in a murine Matrigel sponge implant model as well as a murine tumor model. These results suggest a role for mGluR1 in breast cancer as a pro-angiogenic factor as well as a mediator of tumor progression. They also suggest mGluR1 as a potential new molecular target for the anti-angiogenic therapy of breast cancer.

## Introduction

Angiogenesis is critical for normal physiological processes, including wound healing, embryonic development, and the menstrual cycle. Tumors are also critically dependent upon their ability to hijack the normal physiologic process of angiogenesis and thereby induce the ingrowth of blood vessels from the host in order to grow, invade, and metastasize [1], [2]. The process of angiogenesis is normally tightly regulated through control of the relative levels of pro- and antiangiogenic factors, a process that has been described as the “angiogenic balance” [3], [4]. However, malignant cells can shift the angiogenic balance away from homeostasis towards angiogenesis through the secretion of proangiogenic factors, the most common of which is VEGF [5], a peptide growth factor secreted by a wide variety of cancers, beginning early in progression [6]. Numerous studies have reported a correlation between increased angiogenesis and poor prognosis in various cancers [7], [8], and inhibiting tumor-induced angiogenesis has emerged over the last decade as a promising strategy for cancer therapy. Indeed, the combination of antiangiogenic therapy with conventional therapies, in particular radiation therapy and cytotoxic chemotherapy, has led to significant increases in overall survival in certain cancers such as colorectal cancer metastasis to the liver [9]. However, antiangiogenic therapy is not without its drawbacks. For example, bevacizumab, a humanized mouse monoclonal antibody to VEGF that is currently the most commonly used antiangiogenic therapy for cancer, is expensive, must be given intravenously, and produces side effects of hypertension, hemorrhage and even intestinal perforation, among others [10], [11]. In addition, tumors can overcome bevacizumab by producing more VEGF, leading to resistance. [11].

Of the downstream mediators of VEGF receptors, PKC is known to be a crucial mediator [12], [13]. In a previous study, Riluzole, a known inhibitor of PKC activity [14], has been shown to mediate endothelial cell (EC) proliferation and abnormal vessel formation in a rat model of retinopathy [15]. In addition to its well known inhibitory effect on PKC, Riluzole also mediates other signaling pathways including mGluR1-mediated glutamate release [16], [17] suggesting a role for mGluR1 in mediating angiogenesis. Glutamate signaling occurs through binding to ionotropic or metabotropic receptors (mGluRs). mGluRs (genes: *GRM1*-*GRM8*; protein receptors: mGluR1-mGluR8) belong to the family of G-protein-coupled seven transmembrane domain receptors (GPCRs) [18], which mediate responses to a diverse array of signaling molecules, including hormones, neurotransmitters and chemokines which can act in an autocrine or paracrine manner [19]–[22].

In the mammalian CNS, mGluRs are categorized into either group I, II, or III receptors based on sequence homology, agonist selectivity, and effector coupling. They are essential for normal neuronal function and have been implicated in a wide range of neurological disorders including amyotrophic lateral sclerosis (ALS) [23]–[25], Parkinson's disease [26], and depression [27], in addition to various cognitive disorders [26]. mGluR1 and mGluR5 comprise Group I and are mainly involved in excitatory responses induced by strong presynaptic stimulation [28]–[30]. They are coupled to a Gαq-like protein and activate signaling cascades known to be involved in proliferation [27], [31]. In addition, group I mGluR activation modulates a variety of ion channels including the L-type voltage dependent calcium channels [32]–[34] and activates a wide range of protein kinase pathways (PKA, CaMKs, MAKs, PI3K) which link mGluRs to transcriptional changes within a cell [27], [31], [34], [35]. Within the past decade, mGluR expression, in particular that of mGluR1, has been detected in brain endothelial cells where mGluRs appear to play a protective role in response to various insults such as hypoxia, glutamate, and NO-induced vascular injury [36], [37]. Based on these studies and our recent finding demonstrating inhibition of tumor progression by Riluzole and the mGluR1 inhibitor, BAY36-7620 [38], we hypothesize that mGluR1 activity may play a key role in regulating EC phenotype during tumor-induced angiogenesis and therefore might represent a molecular target for the antiangiogenic therapy of cancer. In this paper, we test this hypothesis and demonstrate that a loss of mGluR1 expression and activity is associated with an anti-angiogenic phenotype and tumor suppression.

## Materials and Methods

### Reagents and cell culture

All EC culture reagents and human dermal microvascular endothelial cells (HDEC) were purchased from Lonza (Walkersville, MD). Primary human umbilical vein endothelial cells (HUVEC) and human pulmonary microvascular endothelial cells (HLEC) were purchased from ScienCell Research Laboratories (San Diego, CA). The immortalized human dermal microvascular endothelial cell line (HMEC-1) was obtained from the Centers for Disease Control and cultured as described [39]. All ECs were cultured in their respective EGM-2 medium (basal medium) containing the appropriate supplements and 10% serum which were all purchased from Lonza and primary ECs were only used for experimental analyses up to ten passages. The mouse mammary carcinoma cell line 4T1-12B was a kind gift from Fred Miller (Karmanos Cancer Institute) and MDA-MB-231 cells were purchased from ATCC. Both cell lines were maintained in RPMI containing 10% serum and 1% penicillin/streptomycin purchased from Invitrogen-Life Technologies (Carlsbad, CA).

Reagents for immunohistochemical analysis, including secondary antibodies, were purchased from either Vector Laboratories (Burlingame, CA) for sponge sections or from Santa Cruz (Santa Cruz, CA) for tumor sections. mGluR1 inhibitors BAY36-7620 and YM 298198 were purchased from Tocris (Norwich, UK), and research grade Riluzole was purchased from Sigma-Aldrich (St. Louis, MO). Antibody against mGluR1 was purchased from Millipore (Temecula, CA) and antibodies against human and mouse CD31 were purchased from Abcam (Cambridge, MA) and Santa Cruz Biotechnology, Inc. (Santa Cruz, CA), respectively. Western analysis reagents were purchased from Bio-Rad (Hercules, CA) and 3-(4,5-dimethylthiazol-2-yl)-2,5-diphenyltetrazolium bromide (MTT) was purchased from Invitrogen-Life Technologies.

### mGluR1 Protein Expression

mGluR1 expression in ECs was measured by Western blot analysis. Briefly, cells were collected by scraping in RIPA lysis buffer (Santa Cruz) containing 10 mM Tris-HCl, 1% Nonidet P-40, 0.5% sodium deoxycholate, 0.1% SDS, 0.004% sodium azide, and supplemented with a protease inhibitor cocktail solution. 75–100 ug of protein was separated by SDS-polyacrylamide gel electrophoresis (4–20%) and transferred to polyvinylidene fluoride membranes. Immunodetection of mGluR1 was performed using either rabbit polyclonal anti-mGluR1 antibody (Millipore) or mouse monoclonal anti-mGluR1 antibody (BD Biosciences, Bedford, MA) with appropriate secondary antibodies and detected by chemiluminescence. Primary blots were stripped and reprobed with antibody against GAPDH (Novus Biologicals, Littleton, CO).

### RT-QPCR analysis of *GRM1* expression

Total RNA was extracted from ECs using RNeasy Plus Mini Kit (Qiagen, Valencia, CA) according to manufacturer instructions. Reverse transcription was performed with 2 ug RNA using High-capacity cDNA Reverse Transcription Kit (Applied Biosystems-Life Technologies) according to the manufacturer's instructions. QPCR was performed using ABsolute QPCR SYBR Green Mix (Thermo Scientific) and oligonucleotide primers for *GRM1* and GAPDH, as described previously [40]. Thermal cycling was performed under the following conditions: 15 min enzyme activation step at 95°C followed by 35 cycles of denaturation (15 sec at 95°C), annealing (30 sec at 60°C), and extension (30 sec at 72°C). No-RT controls were used to confirm lack of contaminating genomic DNA.

### *GRM1* transduction assays

Lentiviral particles containing GRM1 shRNA vectors or non-silencing control vector DNA (Thermo Scientific-Open Biosystems), were generated by reverse transfection of these constructs, together with Trans-Lentiviral package mix, into HEK293T cells using Arrest-In/Express-In transfection reagent. Approximately 10^6^ TU/ml was used to infect HUVEC in the presence of polybrene (10 ug/ml) and a stable culture was generated by growing these cells in the presence of 1 ug/ml puromycin, the lowest concentration observed to kill 100% of non-transduced HUVECs (data not shown). All reagents for these transduction assays were purchased from Thermo Scientific.

### Cell Proliferation

To determine a role for mGluR1 signaling on cell growth, various ECs were plated at 1×10^5^ cells/well into 96-well plates in EBM-2 basal medium (no supplements) in reduced serum (5%) plus 100 ng/ml VEGF (R&D systems, Minneapolis, MN) and exposed to various mGluR1 inhibitors, or vehicle (0.05% DMSO). Proliferation was determined once a day for three days by measuring the conversion of water soluble MTT into an insoluble formazan product. Briefly, 12 mM MTT (Invitrogen-Life Technologies) was added to the wells and allowed to incubate for 2–4 hours at which time DMSO was added to the wells to lyse the cells and dissolve the formazan. The formazan product was detected by measuring absorbance at 540 nm and results expressed as % of control (no VEGF) where no growth was demonstrated. In some experiments, cell numbers were also determined in parallel with the MTT assay by counting manually on a hemacytometer. The results of the inhibitor studies were confirmed in a second set of experiments using sh*GRM1*-infected HUVECs.

### Endothelial tube formation assays

ECs were plated on reduced serum Matrigel basement membrane matrix (BD Biosciences) at either 1×10^5^ cells/ml (HUVEC) or 2.5×10^5^ cells/ml (HMEC-1) in 24 well plates and incubated overnight in basal EGM-2 media containing FBS (1%), VEGF (100 ng/ml) and in the presence and absence of increasing concentrations of either BAY36-7620 or Riluzole. After 18–24 hours, brightfield images (using a 4× objective), were taken to capture the entire well (4 image fields/well) using a Nikon Eclipse TE2000-U inverted microscope and the endothelial tubes formed were counted using the Java-based image processing program (ImageJ64) developed by the National Institutes of Health. Results were expressed as the average tubes formed/field and compared to vehicle treated cells (0.05% DMSO). The results of the inhibitor studies were confirmed in a second set of experiments using sh*GRM1*-infected HUVECs.

### Matrigel sponge model

Angiogenesis was measured in vivo using the Matrigel sponge model originally developed by Dr. Nor at the University of Michigan [40]. Briefly, porous poly-l-lactic acid (PLLA) sponges were prepared by dissolving PLLA in chloroform to yield a 5% solution and then 1.6 ml of this solution was combined with 2.3 g of sodium chloride in a beaker pre-treated with silicone. The chloroform was evaporated and the sponges formed were washed 5 times over a two-day period to remove the salt and stored under vacuum suction until use. The day before implantation, the sponges were cut into 10×10×1 mm thick squares and then sterilized by soaking in 100% ethanol for 2 hours followed by 2 washes of PBS and then left overnight in PBS. On the day of implantation, HDECs were resuspended in Matrigel basement membrane matrix and EBM-2 medium with supplements and 10% FBS at a ratio of 1∶1 and 36 ul containing 1×10^6^ cells were placed on top of each sponge and allowed to soak in for a few minutes. The sponges were placed into the flanks of 15 female athymic nude (nu/nu) mice, aged between 6 and 8 weeks (Harlan Laboratories, Indianapolis, IN), divided into groups of 5 and treated i.p. starting the next day with either BAY36-7620 (10 mg/kg), Riluzole (18 mg/kg) or vehicle (DMSO). Treatment continued once a day for 14 days at which time the mice were euthanized and the sponges removed and placed in 10% formalin for vessel analysis. Animals were housed in a pathogen-free facility and all animal studies were performed in accordance with the recommendations in the Guide for the Care and Use of Laboratory Animals of the National Institutes of Health. The protocol was approved by the local IACUC at Wayne State University School of Medicine, Detroit, MI (Protocol Number: 03-03-11). Surgery was performed using carprofen analgesic and under ketamine/xylazine anesthesia and all efforts were made to minimize suffering.

### Syngeneic breast tumor model

4T1 cells (3×10^4^) were injected into the #4 mammary fat pads of female BALB/c mice, aged between 6 and 8 weeks old (Harlan Laboratories), and allowed to grow for 10 days at which time small tumors had begun to grow (average size of 62 mm^3^). Mice were then divided into groups of 10 and treated with daily i.p. injections of Riluzole (18 mg/kg), Sunitinib (20 mg/kg), or vehicle (DMSO) for 14 days. Tumor size was measured two to three times a week using a Vernier caliper and tumor volume was calculated using the following formula: length × width × depth/2. Animals were housed in a pathogen-free facility and all animal studies were performed in accordance with the recommendations in the Guide for the Care and Use of Laboratory Animals of the National Institutes of Health. The protocol was approved by the local IACUC at Wayne State University School of Medicine, Detroit, MI (Protocol Number: 03-02-11).

### Immunohistochemistry

Sponges and tumor samples were placed in 10% formalin, paraffin-embedded and sectioned. To stain, sections were first deparaffinized using two changes of xylene for 3 minutes and rehydrated through a graded series of ethanol (100%, 95%, 70%, 50%) to water. Sodium citrate was used for antigen retrieval and sections were blocked in 5% serum from the host of the secondary antibody. Microvessel density was determined by incubating sections in either rabbit polyclonal anti-CD31 (Abcam) for sponge sections or goat polyclonal anti-CD31 (Santa Cruz) for tumor sections followed by incubation in the appropriate FITC-conjugated secondary antibodies. Slides were cover-slipped using SlowFade Gold antifade reagent with DAPI (Invitrogen) and examined on a Nikon Ti E-Series inverted microscope. Fluorescent images were captured under identical conditions where fluorescent intensity was adjusted to exclude background signal from the isotype-containing secondary antibody. Sponges were evaluated for vessel formation by immunofluorescence where CD31-expressing vessels were counted and averaged. In this angiogenic model, vessels are not homogenously distributed throughout the sponges. Instead, they occur in groupings or “hotspots” with at least 5 hotspots occurring per treatment slide. Therefore, to interpret the effect of Riluzole and BAY36-7620 in this model, vessels were counted in 5 hotspot fields per slide (2 slides per animal) using ImageJ64 and results expressed as the average number of vessels per field (200×). Tumors were evaluated for microvessel density by counting and averaging the number of vessels per tissue area (mm2) using ImageJ64. CD31 stained cells were considered a vessel when a structure contained more than 2 CD31 positive-stained cells and structures that formed parts of the same vessel were counted as a single vessel.

### Statistical analysis

Data were analyzed using GraphPad Prism version 4 for Macintosh (GraphPad Software, San Diego, CA). All numerical results are expressed as mean ± SEM and statistical analysis was performed by either one-way or two-way repeated-measures analysis of variance (ANOVA) followed by a multiple comparison procedure with the Student-Newman Keuls method. A value of ≤0.05 was considered significant.

## Results

### mGluR1 is expressed in human endothelial cells and regulates cell proliferation

**Figure 1:**
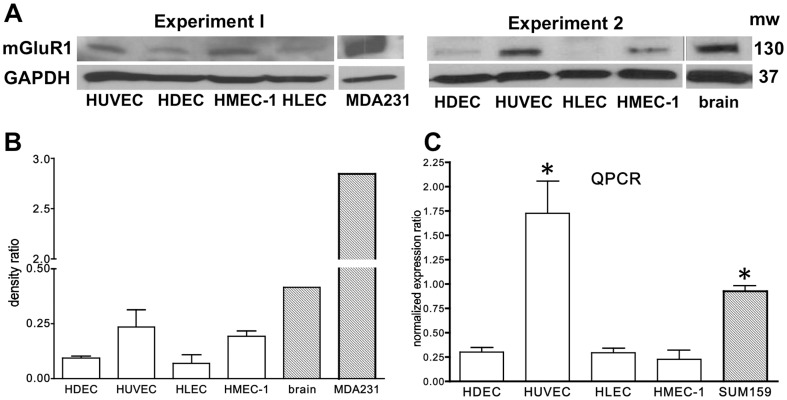
mGluR1 and *GRM1* expression in various human vascular endothelial cells. **A**. mGluR1protein was detected by Western analysis in primary human dermal microendothelial cells (HDEC), human umbilical vein endothelial cells (HUVEC), human lung microendothelial cells (HLEC), as well as in the human microendothelial cell line (HMEC-1). Human brain lysate or MDA-MB-231 (MDA231) cells overexpressing *GRM1* by Lentiviral transduction were used as a positive control for mGluR1 and GAPDH was used as a loading control. **B.** Combined density graph of (**A**), normalized to their respective GAPDH controls. **C**. *GRM1* message was detected in HDEC, HUVEC, HLEC, and HMEC-1 by qPCR and normalized using GAPDH as the reference gene. *GRM1* message is detected in all ECs tested, comparable to levels detected in the breast cancer cell line (SUM159). These results represent the mean ± SEM of two experiments, performed in triplicate where * is p<0.05 compared to HDEC, HLEC, or HMEC-1.

**Figure 2:**
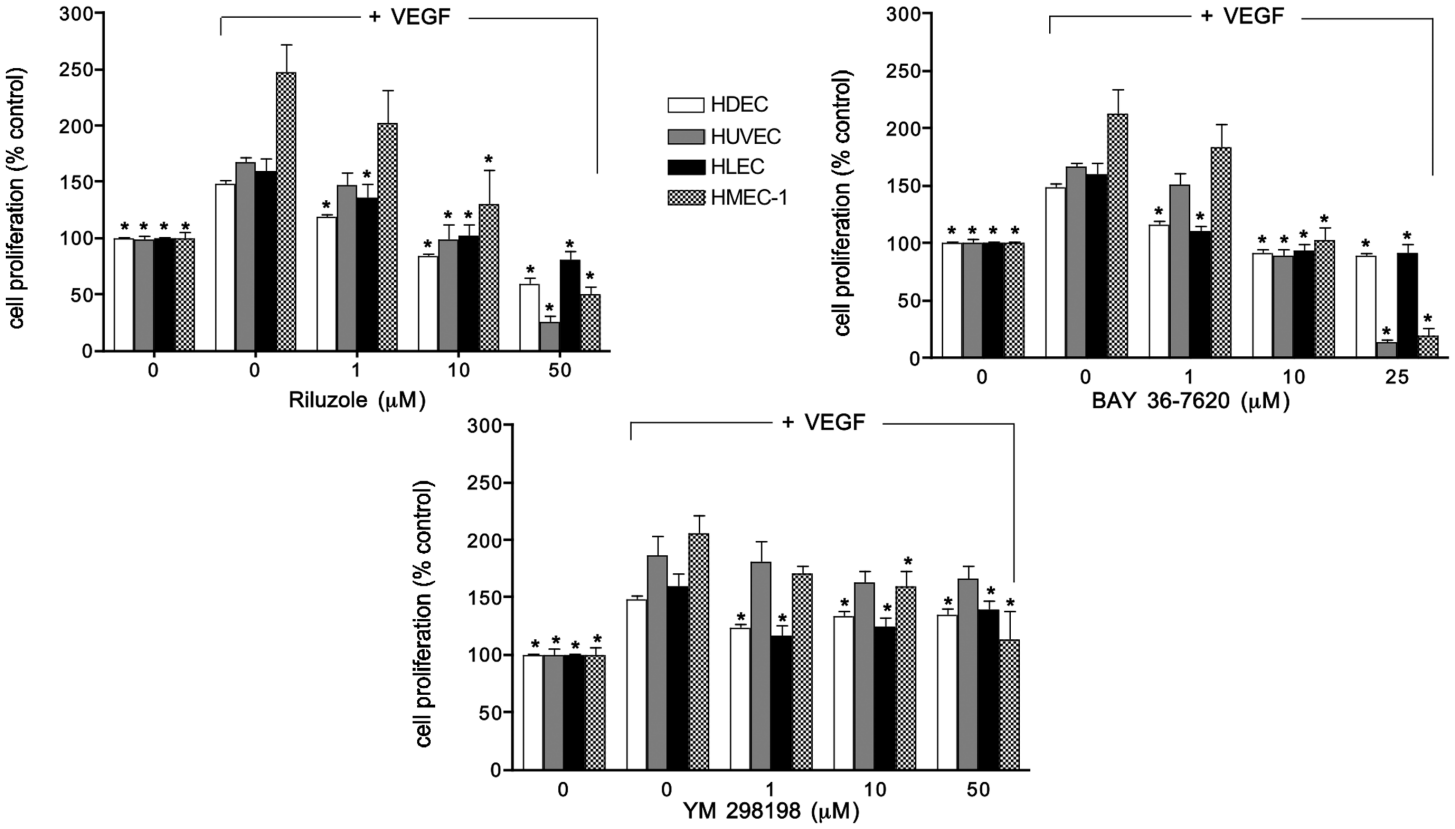
Inhibition of cell proliferation by mGluR1 antagonists. Human dermal microendothelial cells (HDEC), human umbilical vein endothelial cells (HUVEC), human lung microendothelial cells (HLEC) and the human microendothelial cell line (HMEC-1) were plated at 1×10^5^ cells/well into 96-well plates in EBM-2 basal medium (no supplements) in reduced serum (5%) containing 100 ug/ml VEGF and exposed to various mGluR1 antagonists, or vehicle (0.05% DMSO). Cell proliferation was determined on day 3 by MTT assay where formazan product was detected by measuring absorbance at 540 nm and results expressed as % control (no VEGF) where no growth was demonstrated. Both Riluzole (**A**) and BAY36-7620 (**B**) significantly inhibited cell proliferation in a dose-response manner in all EC types whereas YM 298198 (**C**) had a significant effect on cell proliferation in all the cell types except HUVEC. Results are the mean ± SEM of three experiments, performed in triplicate, where * is p<0.05 compared to their respective control cells containing VEGF.

To determine whether mGluR1 is expressed in human ECs, we assessed several primary ECs (HDEC, HUVEC, HLEC) or cell lines (HMEC-1) for mGluR1 by Western blot analysis. mGluR1 was detected in all four EC types with higher levels expressed in HUVEC and HMEC-1 (Fig. 1A–B). However, these levels were not significantly different from each other. These results were similar to *GRM1* QPCR results where *GRM1* expression levels were demonstrated in all ECs tested with significantly higher levels demonstrated in HUVEC (Fig 1C). Using these ECs we assessed cell proliferation in the presence of the mGluR1 non-competitive antagonists BAY36-7620 and YM298198, as well as Riluzole, at various concentrations for up to 3 days. BAY36-7620 and YM298198 are specific antagonists of mGluR1 that exhibit their effect through direct association with the receptor [41], [42]. Riluzole, in addition to inhibiting mGluR1, has been shown to affect other signaling pathways as well, including but not limited to calcium release, PKC, and voltage-gated sodium channels [43]. We chose to test Riluzole despite its significant off-target effects because, unlike BAY36-7620 and YM298198, Riluzole is FDA-approved for another indication, amyotropic lateral sclerosis, and could therefore be rapidly translated to clinical trials through off-label use as an antiangiogenic agent. In addition, Riluzole shows minimal toxicity with long-term oral administration [44]. Both Riluzole and BAY36-7620 significantly inhibited cell proliferation in all the ECs tested compared to vehicle treated control cells containing VEGF (Fig. 2). Both HUVEC and HMEC-1 were the most sensitive to inhibition by either Riluzole (85% and 70%, respectively) or BAY36-7620 (90% and 91%, respectively) at the highest concentration tested, which is not surprising given that they express the highest level of mGluR1 protein (Fig. 1). YM298198 was also able to inhibit cell proliferation of all ECs tested, however, this effect was not as robust and was only significant in three of the ECs, excluding HUVECs. Since HUVEC express the highest level of mGluR1, these results suggest YM298198 to be a weaker antagonist of mGluR1 compared to Riluzole or BAY36-7620.

**Figure 3:**
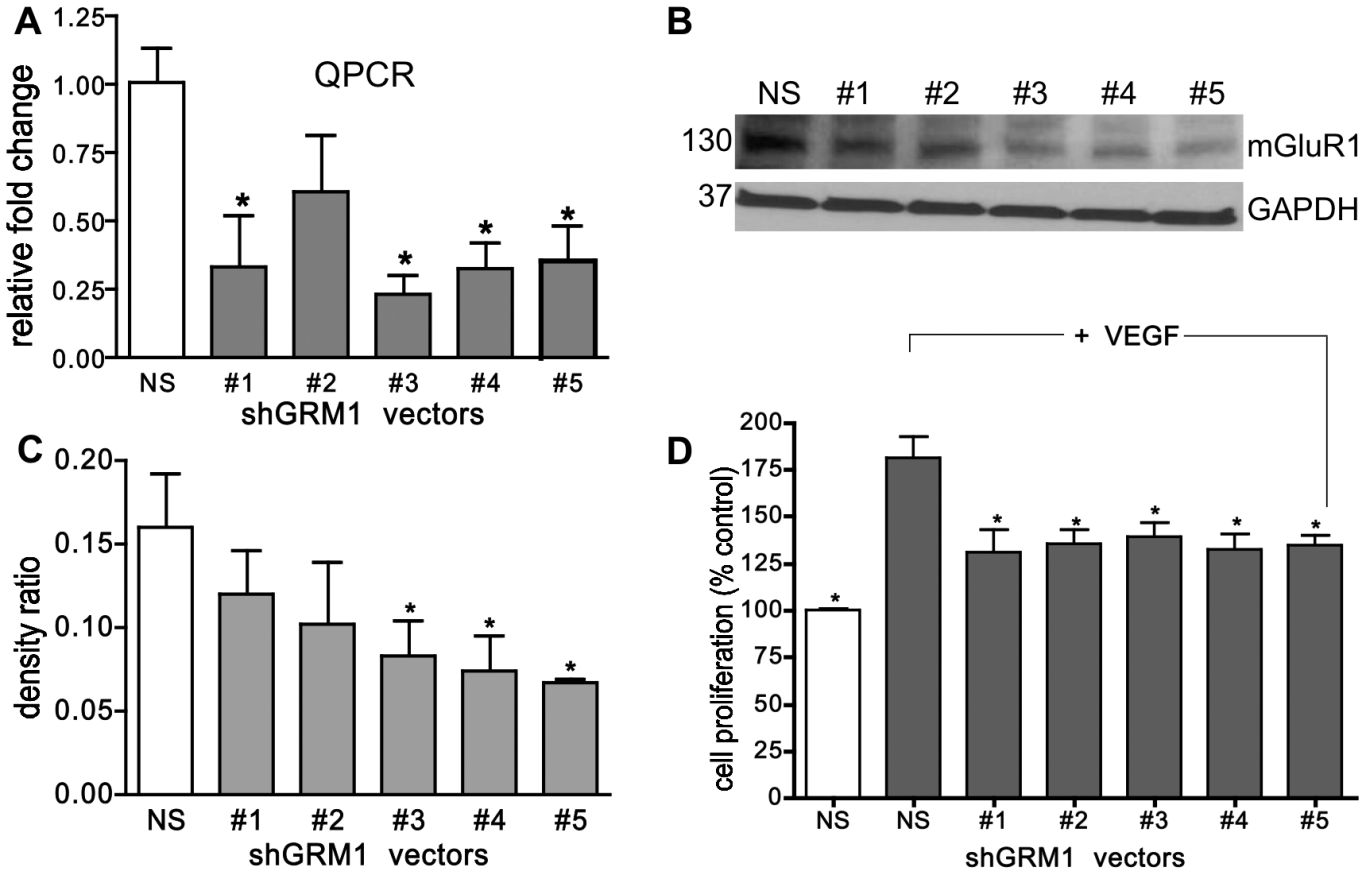
shRNA-mediated knockdown of *GRM1* inhibits cell proliferation of HUVECs. Knockdown of *GRM1* was accomplished by infecting with GIPZ shRNA Lentiviral vectors containing a puromycin resistance gene and either shRNA constructs #1-5 against *GRM1* or a non-silencing shRNA construct (NS). Cells were selected with puromycin (1 ug/ml) for 10 days and *GRM1* message (**A**) or mGluR1 protein levels (**B**) were determined by QPCR or Western analysis, respectively. **C**. Density graph of (**B**) repeated twice and normalized to their respective GAPDH controls. **D**. The effect of *GRM1* knockdown on the proliferation of HUVECs was determined 10 days post-infection by MTT assay. The sh*GRM1* infected cells were plated 10 days after infection in 96-well plates at 1×10^5^ cells/well into 96-well plates in EBM-2 basal medium (no supplements) in reduced serum (5%) plus 100 ng/ml VEGF and cell proliferation was determined on day three. These results are representative of two *GRM1* silencing experiments performed in triplicate where * is p<0.05 compared to NS-infected VEGF-treated cells.

To confirm a role for mGluR1 in mediating cell proliferation, HUVECs were transduced with Lentiviral vectors expressing one of five *GRM1* silencing shRNAs or a non-silencing control vector (NS) and after which they were stably selected using puromycin (1 ug/ml) and then cell proliferation was measured. According to Western and QPCR analysis, ten days after infection, both mGluR1 protein levels and *GRM1* message were inhibited in all sh*GRM1*-infected cells compared to NS vector-treated cells (Fig. 3A–C). *GRM1* message was significantly inhibited by a maximal 60–75% in the HUVECs infected with all the sh*GRM1* vectors except # 2 (Fig. 3A). Interestingly, there was a strong association between the potency of vectors #3-5 at knocking down *GRM1* message with its ability to inhibit mGluR1 protein expression (Fig. 3B–C). Cell proliferation, which was assessed 10 days post-infection, was significantly inhibited in all the sh*GRM1*-silenced cells with maximal inhibition averaging around 50%, compared to the NS VEGF-treated cells (Fig. 3D).

### mGluR1 regulates Matrigel tube formation in HUVEC and HMEC-1

**Figure 4:**
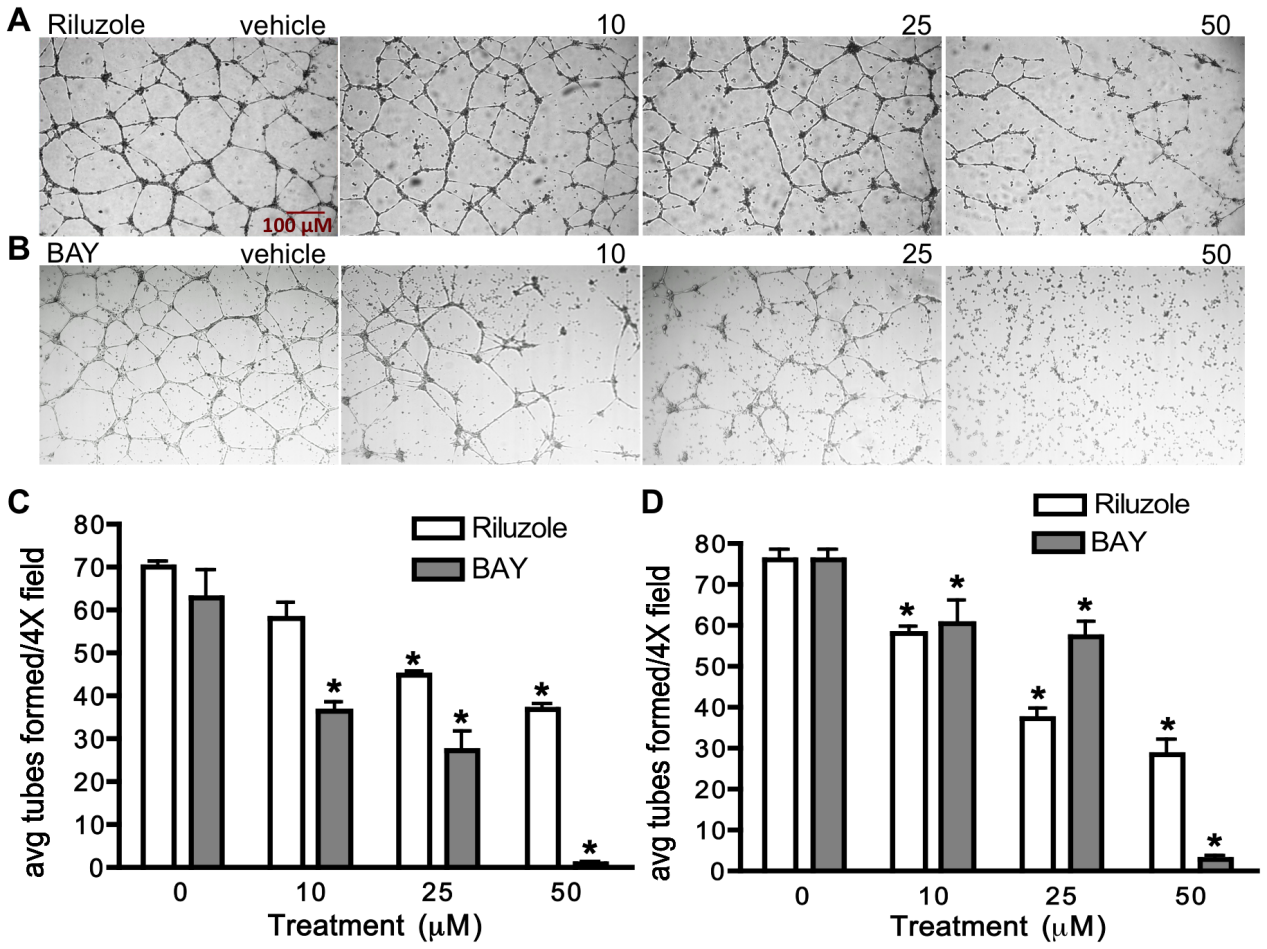
Riluzole and BAY36-7620 inhibit endothelial tube formation on Matrigel. HUVEC and HMEC-1 were plated onto Matrigel coated 24 well plates at 2×10^5^ cells per well and incubated overnight in the presence of medium containing FBS (1%), VEGF (100 ng/ml) and either 0 (vehicle), 10, 25, or 50 uM Riluzole or BAY36-7620 (BAY) after which cells were photographed and the number of tubes per 4× field were counted and averaged. Both Riluzole (**A**) and BAY (**B**) inhibited tube formation in HUVECs in a dose-dependent manner compared to vehicle (DMSO) treated cells. **C** and **D** represent the results of two experiments performed in triplicate in HUVECs and HMEC-1, respectively where * is p<0.05 compared to vehicle (DMSO) treated cells.

**Figure 5:**
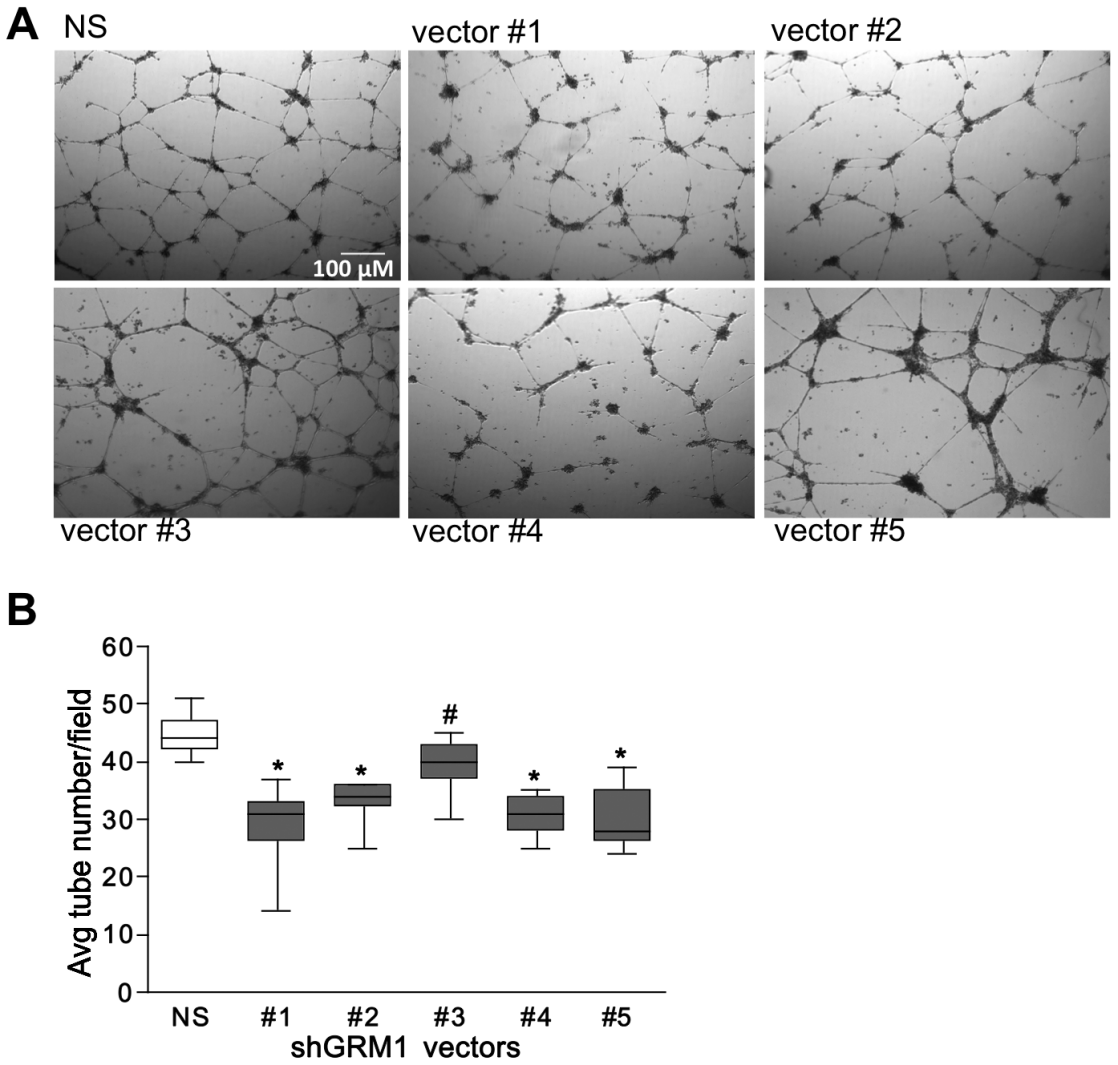
shRNA-mediated knockdown of *GRM1* inhibits endothelial tube formation on Matrigel. HUVECs, in which the *GRM1* gene was silenced using GIPZ sh*GRM1* Lentiviral vectors 1–5 or a non-silencing vector (NS) as described previously (Fig. 3 and Methods section), were plated onto Matrigel at 2×10^5^ cells per well and incubated overnight in the presence of medium containing FBS (1%) and VEGF (100 ng/ml). After overnight treatment, cells were photographed (**A**) and the number of tubes formed per 4× field were counted using ImageJ64 software and averaged (**B**). Tube formation on the Matrigel was significantly inhibited in the HUVECs infected by all the sh*GRM1* vectors when compared to HUVECs infected with the NS vector. Results are representative of two experiments performed in triplicate where * is p<0.01 and # is p<0.05 compared to NS-infected cells.

To assess whether mGluR1 mediates angiogenesis, we measured the effect of Riluzole and BAY36-7620 on tube formation by HUVECs and HMEC-1 cells when plated on serum-reduced Matrigel in the presence of VEGF. We chose these two inhibitors because of their ability to inhibit cell proliferation in these cells. The ability of HUVEC (Fig 4A–C) and HMEC-1 (Fig. 4D) to form tubes on Matrigel was significantly inhibited in a dose-response manner by Riluzole (48% and 60%, respectively) and BAY36-7620 (98% and 96%, respectively) at the highest concentrations tested. Interestingly, BAY36-7620, which is a more specific inhibitor of mGluR1, demonstrated a greater inhibitory effect on tube formation than Riluzole in both HUVECs and HMEC-1 cells. These results suggest that mGluR1 plays a role in mediating the angiogenic process. To further confirm this role, we repeated the Matrigel tube formation assay using the same shRNA-infected HUVECs as described above (Fig. 5A). Tube formation was significantly inhibited in all the sh*GRM1*-infected cells when compared to NS-infected control, with a maximal inhibitory effect of approximately 35% in all the sh*GRM1*-infected cells except #3 (Fig. 5B). All of the tube formation experiments were performed in the presence of low serum (1%) and 100 ug/ml VEGF (required for tube formation) and MTTs were performed in parallel to confirm any possible effect of cell growth on tube formation.

### mGluR1 inhibits the angiogenic process in vivo

**Figure 6:**
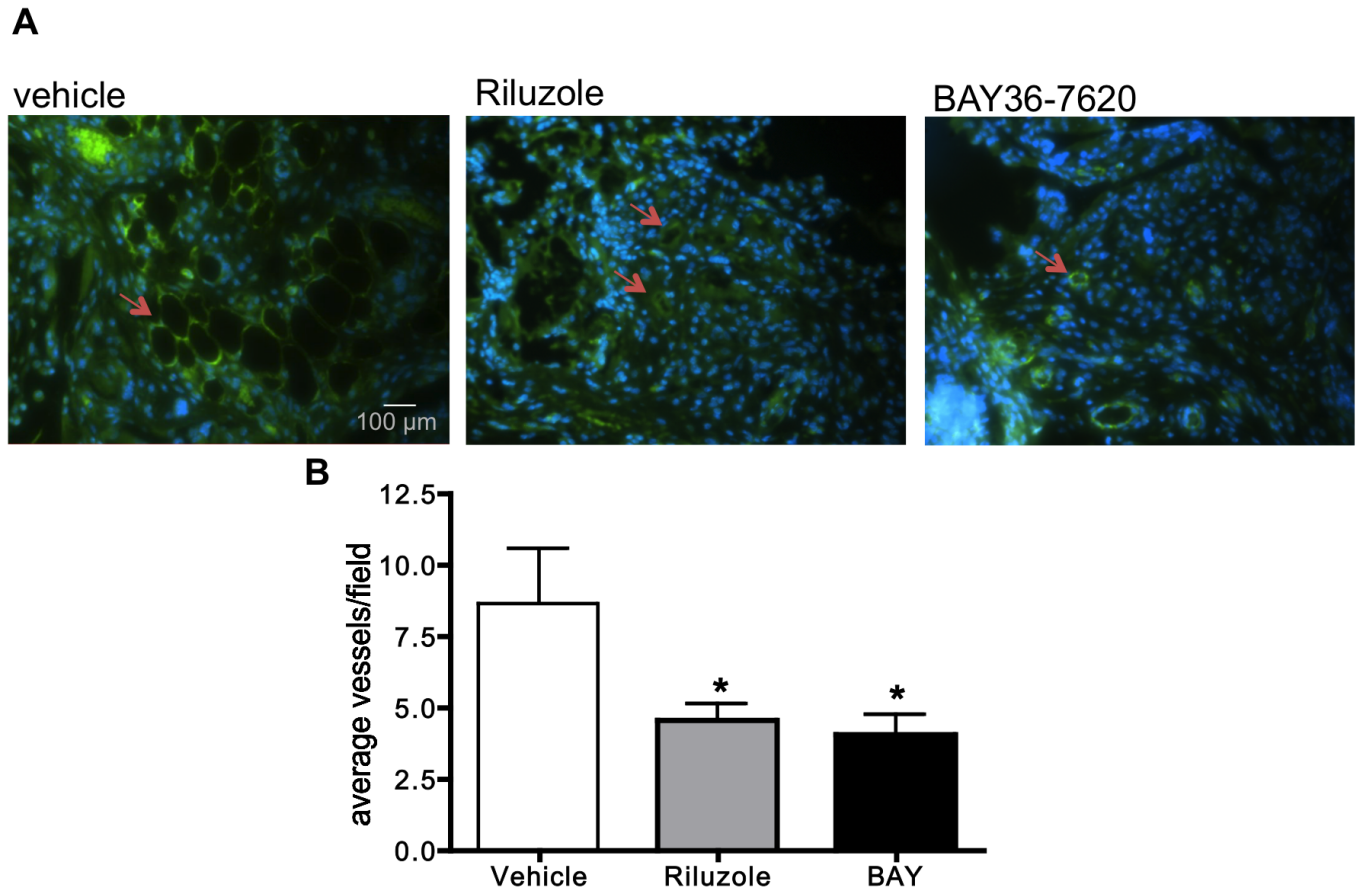
Riluzole and BAY36-7620 (BAY) inhibit angiogenesis in a Matrigel sponge assay. Polylactic sponges containing 1×10^6^ human dermal microendothelial cells were implanted subcutaneously into the flanks of athymic nude (nu/nu) mice. The next day, dosing i.p. with Riluzole (18 mg·kg^−1^·d^−1^) or BAY (10 mg·kg^−1^·d^−1^) was begun and continued for 2 weeks before harvesting. **A**. Vessels were identified by immunostaining using anti-CD31 antibody (green) and counterstained with DAPI (blue). Arrows indicate an average size vessel in each group. **B**. The number of CD31-expressing vessels in 5 hotspot fields per slide (2 slides per animal) was determined using ImageJ64 and the results expressed as the average number of vessels per field. Both Riluzole and BAY significantly inhibited vessel formation by approximately 50% compared to vehicle (DMSO) treated animals where * is p<0.05 compared to vehicle treated mice and n = 5 mice per treatment group.

To determine whether mGluR1 can mediate angiogenesis in vivo, we utilized the Matrigel sponge model originally developed by Nor and Polverini [40]. Matrigel-containing human dermal microendothelial cells were seeded into porous matrices (sponges) and implanted subcutaneously into the flanks of immunodeficient (nude) mice, which were then treated with Riluzole, BAY36-7620, or vehicle (DMSO). After two weeks of treatment, when maximum vessel formation is known to occur [40], the sponges were harvested and the vessels stained with CD31 to visualize for counting. Even though vessel count was relatively low, we were able to detect a significant reduction in the number and size of the vessels formed in the sponges from the inhibitor-treated mice compared to the vehicle-treated mice. In the sponges from both the Riluzole and BAY36-7620 treated mice, vessel formation was inhibited by approximately 50% when compared to the vehicle-treated mice (Fig. 6).

**Figure 7:**
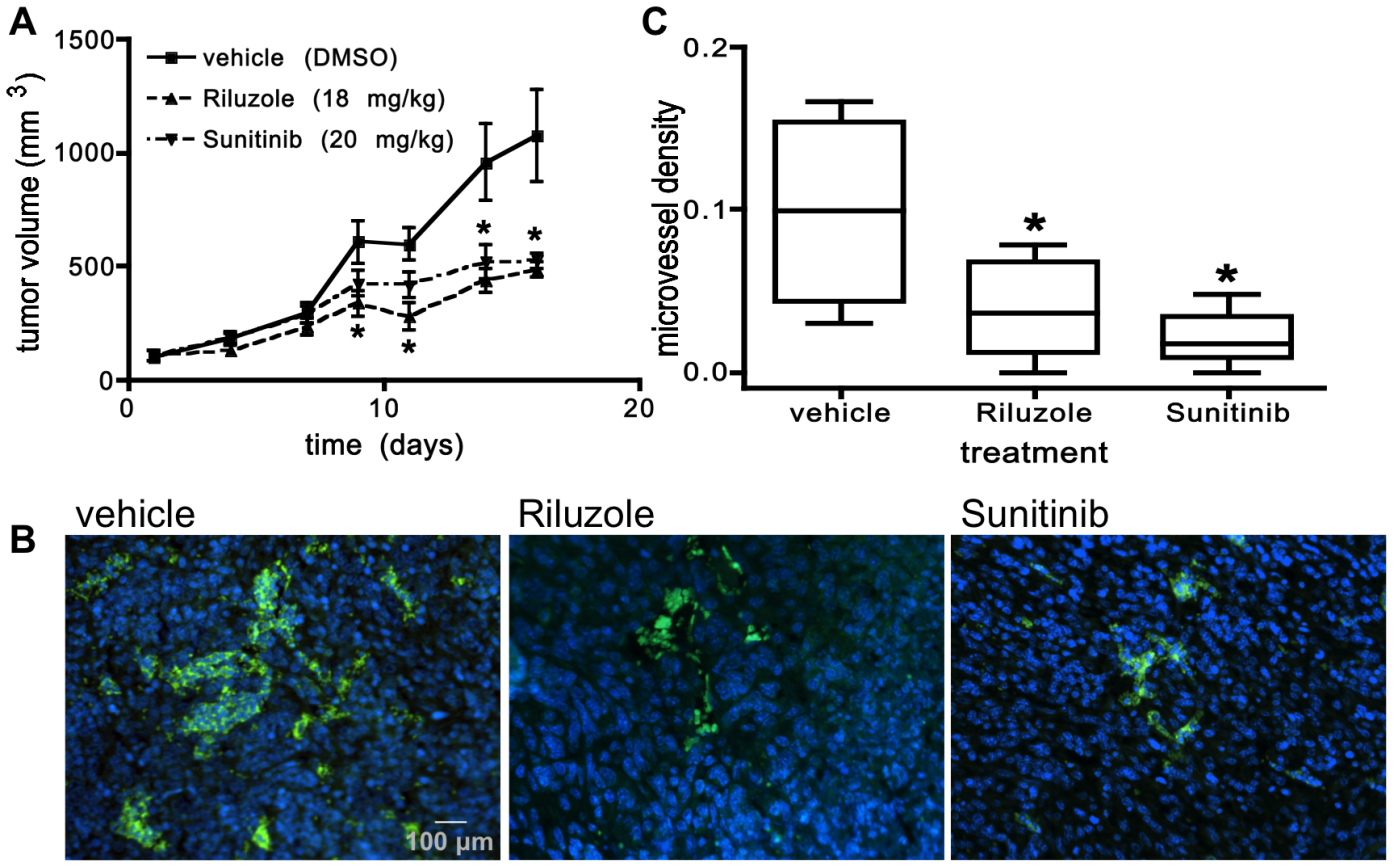
Riluzole inhibits tumor growth and reduces microvessel density in the 4T1 syngeneic breast tumor model. 4T1 breast cancer cells (3×10^4^) were injected into the mammary fat pad of BALB/c mice. Treatment with either Riluzole (18 mg·kg^−1^·d^−1^), Sunitinib (20 mg·kg^−1^·d^−1^), or vehicle (DMSO) was initiated in established tumors (62 mm^3^) on day 10 post tumor cell injection and continued for 14 days. Tumor volumes were measured two or three times weekly and mean tumor volume ± SEM was determined (**A**). * is p<0.05 and n = 10 mice per treatment group. **B.** Tumor sections analyzed for microvessel density by immunofluorescent staining using anti-CD31 antibody (green) and counterstained with DAPI (blue) (1000×). **C.** When compared to vehicle-treated mice, microvessel density was significantly inhibited in both the Riluzole and Sunitinib- treated mice. Microvessel density was determined by counting and averaging the number of vessels per tissue area (mm^2^) per slide using ImageJ64 (100× magnification) and as described previously (Methods section) where * is p<0.05 compared to vehicle-treated mice and n = 10 slides per treatment group.

Recently, we have discovered that mGluR1 is expressed and active in breast cancer cells and that Riluzole, at doses equivalent to doses already being used clinically in humans to treat ALS, can significantly inhibit the growth of breast tumor xenografts [38]. Since angiogenesis is known to play an important role in the development and progression of breast cancer, we wanted to estimate the extent to which Riluzole can inhibit the angiogenic process in breast tumors. To test this, we used the 4T1 syngeneic murine mammary tumor model because this model has been demonstrated to have a strong vascular component [45], [46]. We injected 4T1 cancer cells into the mammary fat pads of mice and, 7 days after injection, started dosing the mice with Riluzole, Sunitinib, a known inhibitor of angiogenesis, or vehicle (DMSO). Similar to its effect on tumor progression in the MDA231 xenograft model, Riluzole was able to inhibit tumor progression in the 4T1 model, significantly inhibiting growth by approximately 50% as early as day 9 compared to the vehicle-treated tumors (Fig. 7A). As expected, Sunitinib had a similar effect on tumor growth, significantly inhibiting tumor growth by approximately 50% that reached significance by day 14. By CD31 staining, we were able to visualize the vessels in the tumors from these mice (Fig. 7B–C). Even though vessel count was relatively low in the tumors from the vehicle-treated mice (Fig. 7C), there was a significant reduction in microvessel density demonstrated in both Riluzole and Sunitinib-treated mice. Microvessel density in both the Riluzole and Sunitinib-treated tumors was inhibited by approximately 60% and 80%, respectively compared to vehicle-treated tumors (Fig. 7C). This result suggests that Riluzole, in addition to its direct effect on tumor cell growth [38], might mediate tumor progression through its inhibitory effect on the angiogenic process. Further experiments will be required to determine the relative importance of the antiangiogenic effect of Riluzole compared to its direct effect on ECs or an indirect effect through its action on tumor cells (e.g., inhibiting the release of pro-angiogenic factors).

## Discussion

In this study, we detected expression of mGluR1 by ECs and, using pharmacological inhibitors and gene silencing, demonstrate a novel role for mGluR1 in mediating various steps of the angiogenic process including EC proliferation, Matrigel tube formation and vessel formation in vivo. To our knowledge, this is the first study in which mGluR1 has been shown to play a role in mediating the angiogenic process. In a previous study, Riluzole was shown to inhibit VEGF-stimulated EC proliferation and vessel formation in a rat model of ROP, mediating its effect through PKC [15]. Although Riluzole is widely known to inhibit glutamate release [16], [17], it does not act directly on glutamate receptors. Rather, it is thought to indirectly inhibit glutamatergic signaling, either through inhibition of glutamate release through its action on ion channels or through its ability to inhibit downstream mediators and targets of mGluR1, such as PKC [14], voltage gated sodium and calcium channels [43], [47].

In the present study, mGluR1 gene silencing resulted in endothelial growth inhibition and reduced vessel formation, suggesting a role for mGluR1 as a mediator of angiogenesis. mGluR1 is a member of the Group I mGluR family which, being GPCRs, are primarily coupled to the activation of Gaq/11 proteins which stimulate the PLCβ pathway, resulting in the cleavage of phosphatidylinositol-4,5-bisphosphate into inositol-1,4,5-trisphosphate (IP3) and diacylglycerol (DAG) [27], [31], [34]. Both DAG and IP3 (by stimulating release of calcium from intracellular stores) activate PKC which has also been shown to activate phospholipase D, phospholipase A2 as well as to modulate a variety of ion channels [32], [33]. Since the angiogenic process is highly dependent on VEGF and PKC is a downstream mediator of VEGF activity, it is possible that PKC acts as a coincidence detector, whereby both VEGF and mGluR1 activity are required for its full activation. Indeed, in a previous study in melanoma cells, stimulation of mGluR1 activated PKC epsilon and ERK1/2 [48] suggesting that this isoform may be involved in mediating mGluR1 effects in endothelial cells as well. In addition to PKC, stimulating group I mGluRs in various neuronal populations activates other protein kinases as well, including cAMP dependent protein kinase (PKA), calcium calmodulin-dependent protein kinases (CaMKs), mitogen-activated protein kinases (MAPKs), phosphoinositide 3-kinase (PI3K), mammalian target of rapamycin (mTOR), p70 S6 kinase, casein kinase 1, and cyclin-dependent protein kinase 5 [26], [27], [33], [34], [49]–[52]. In addition, the PKA, CaMKs, MAPKs, and PI3K pathways have been shown to link group I mGluRs to transcriptional changes as well [27], [31], [35]. Therefore, whether mGluR1 is capable of mediating the angiogenic process by modulating VEGF activity, or independently, through activation of other protein kinase pathways including PKA, CaMKs, MAPKs, or PI3K, or both, is unknown and is the current focus of our lab.

We have previously identified mGluR1 receptors in triple negative breast cancer cells and have demonstrated that inhibiting mGluR1 activity with BAY36-7620 or Riluzole, at doses equivalent to doses already being used clinically in human beings to treat ALS, significantly inhibits the growth of MDA-MB-231 xenografts in mice [38]. In the current study, we have also observed that inhibiting mGluR1 activity with Riluzole significantly inhibits the growth of 4T1 tumors and also results in reduced vessel formation, suggesting that mGluR1 can inhibit angiogenesis within the tumor microenvironment. Therefore, it is plausible that mGluR1 plays a dual role in TNBC, both in the tumor compartment, where it directly stimulates tumor cell growth, and in the tumor microenvironment, where it stimulates angiogenesis. The results of this study suggest that mGluR1 represents a promising molecular target in TNBC, an aggressive type of breast cancer with a strong vascular component. Because Riluzole is already an FDA-approved drug with low toxicity and few side effects, the repurposing of Riluzole for the treatment of TNBC may represent a promising therapeutic strategy. It is not difficult to envision using Riluzole in combination with conventional therapy such as chemotherapy and radiation therapy, which we have already shown to work in synergy with VEGF therapy in various cancers [53] or in a role similar to aromatase inhibitors and Tamoxifen in hormone-responsive breast cancers.
